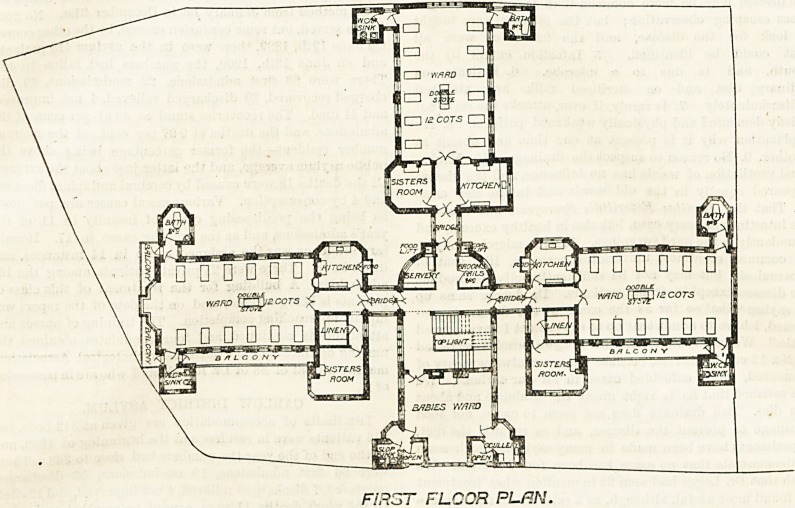# Belgrave Hospital for Children, Clapham Road, S.W.

**Published:** 1901-10-12

**Authors:** 


### The Institutional Workshop.

### BELGRAVE HOSPITAL FOR CHILDREN, CLAPHAM ROAD, S.W.

This hospital was founded in 1866, and ever since that date it has carried out much useful work in its district. The old building could no longer provide sufficient accommodation to meet the calls on it, and moreover it had many structural defects. It was therefore wisely decided to transfer it to the south side of the Thames, and a suitable site was found at the corner of Clapham Road and Prima Road. Here the foundation stone was| laid by the Princess Henry of Battenberg on June 27th, 1900.

The main entrance is in Clapham Road. On the left on entering is an isolation-room having a second door leading into a court. The room can also be entered from the surgery by merely crossing the court. By these means an infectious case can be put outside the hospital at once without passing through any other part of the hospital. The porter's room and secretary's office are close to the entrance. The board room and matron's quarters are on the right of the hall, and balancing these on the left are the surgery and resident medical officer's rooms. At the back of the hall is the kitchen with its various offices. Food lifts run up to the floors above.

The entrance for out-patients is placed in Prima Road, and this department contains a fine waiting-room, consulting-rooms, a room for whooping-cough cases, and an isolation room under the latter. There are a dispensary, and a room where patients can wait until their medicine is prepared. When a patient has been prescribed for and obtained the medicine, he does not again pass through the department, but leaves by another door, and thus confusion is avoided. The out-patients' department is of one story only, except that there are three single rooms and a nurses' room over part of it.

The main staircase is in the hall. On the first floor, and directly over the board-room and matron's quarters, is a ward for 12 cots, a sister's room, a ward kitchen, and properly designed blocks for bath-rooms and closets. In all cases these are cut off from the main by cross-ventilating passages. Each cot has a window on either side of it, except those near the lavatory passages, and there its absence is to a great extent made up for by a large window in the end of the ward which will look well, and be a great help to the ventilation.

The ward is about 40 feet long and 25 feet wide. Assuming the ceilings to be 14 feet high this would give 1,200 cubic feet of air space per cot; an ample allowance, provided an efficient system of ventilation has been adopted. On the latter far more depends than on mere cubic space.

There are two other wards of similar size and construction on this floor, and in addition there is a babies' ward obtained over the main entrance and rooms adjoining. The number of cots in this ward is not stated.

The second floor is similar to the first, but the space over the babies' ward is used as an operating-room, and this room is provided, as every operating-room should be, with direct north top-light

THE BELGRAVE HOSPITAL FOR CHILDREN.
CLAPHAM ROAD. S.W.

GROUND FLOOR PLAN.

Percy Adams Architect 28 Woburn Place W.C.

A very slight examination of the plan will show that any one of these wards could, if necessity arose, be entirely cut off from its fellows, an arrangement of no little importance where children are under treatment. The third floor is for the accommodation of the nursing staff, and the fourth floor is for the domestic staff.

FIRST FLOOR PLAN.

The hospital is built of red brick with Portland stone dressings, and the roofs are tiled.

The elevations are treated with equal boldness and success.

The architect is Mr. H. P. Adams of Woburn Place, Russell Square. The cost is not stated.

## Figures and Tables

**Figure f1:**
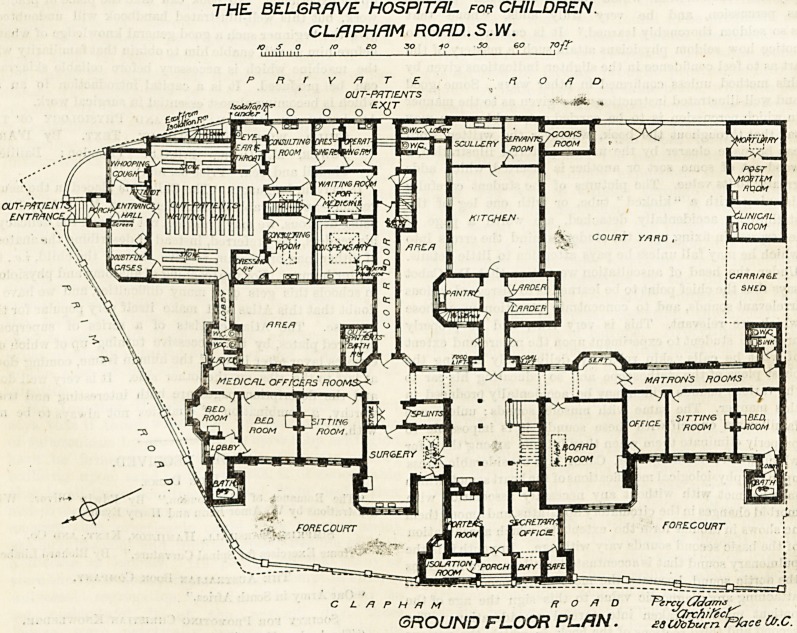


**Figure f2:**